# Environmental pollution effect on honey bees and their derived products: a comprehensive analysis

**DOI:** 10.1007/s11356-024-33754-4

**Published:** 2024-06-07

**Authors:** Rodica Margaoan, Giulia Papa, Alexandru Nicolescu, Mihaiela Cornea-Cipcigan, Mustafa Kösoğlu, Erkan Topal, Ilaria Negri

**Affiliations:** 1 Department of Animal Production and Food Safety, Faculty of Veterinary Medicine, University of Agricultural Sciences and Veterinary Medicine, Cluj-Napoca, Romania; 2 Department of Sustainable Crop Production—DIPROVES, Università Cattolica del Sacro Cuore, Via Emilia Parmense 84, 29122 Piacenza, Italy; 3 Department of Horticulture and Landscape, Faculty of Horticulture and Business in Rural Development, University of Agricultural Sciences and Veterinary Medicine, Cluj-Napoca, Romania; 4 Department of Pharmaceutical Botany, “Iuliu Hațieganu” University of Medicine and Pharmacy, Gheorghe Marinescu Street 23, 400337 Cluj-Napoca, Romania; 5 Apiculture Research Center, Aegean Agricultural Research Institute, 35661 Izmir, Turkey; 6 Izmir Food Control Laboratory Directorate, Bornova, 35100 Izmir, Turkey

**Keywords:** Honey bee, Bee products, Pollution, Health, Risk factors

## Abstract

Several factors, including environmental degradation, air pollution, intense urbanization, excessive agriculture, and climate change, endanger the well-being of animals and plants. One of the major issues with an increasingly negative impact is agricultural contamination with pesticides and antibiotics. Seed coatings with neonicotinoid insecticides used as a protective layer against pests are shown to exceed the permissible limits in most cases. Neonicotinoid compounds bind to nicotinic acetylcholine receptors, therefore affecting the honey bees’ brain. Heavy metals in higher concentrations are lethal for honey bees, and the residue in bee products might pose a threat to human health. Highly effective acaricides used to treat *Varroa destructor* infestations in honey bee colonies have negative effects on honey bee reproduction, olfaction, and honey production. Furthermore, amitraz and fluvalinate are mostly found in the highest amounts and lead to decreased honey production and reduced colony reproduction, along with decreased learning ability and memory. However, scientific studies have shown that honey bees act as a reliable bio-indicator of environmental pollution. In response to the growing demand for bee products, the effects of adulteration and improper storage conditions have gotten worse and represent a new risk factor. In light of the shifting global economy, it is important to analyze consumer expectations and adjust manufacturing accordingly. By ensuring the manufacture of high-quality, traceable products devoid of drug residues, consumers will be better protected from subsequent health problems. This review’s objectives are based on the necessity of identifying the risks associated with honey bees and bee products.

**Graphical abstract:**

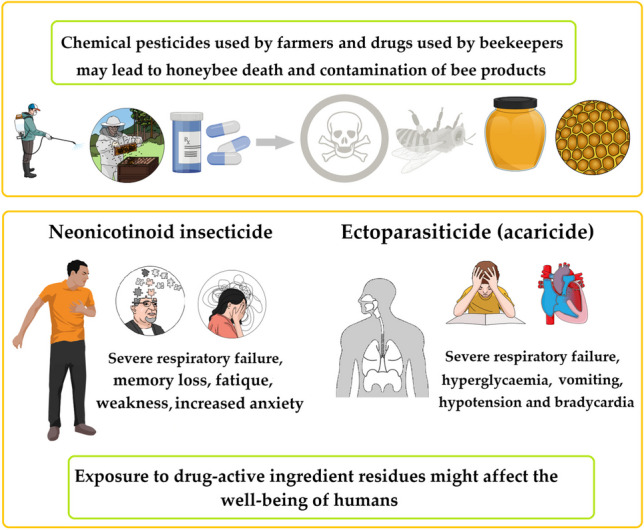

## Introduction

**Fig. 1:**
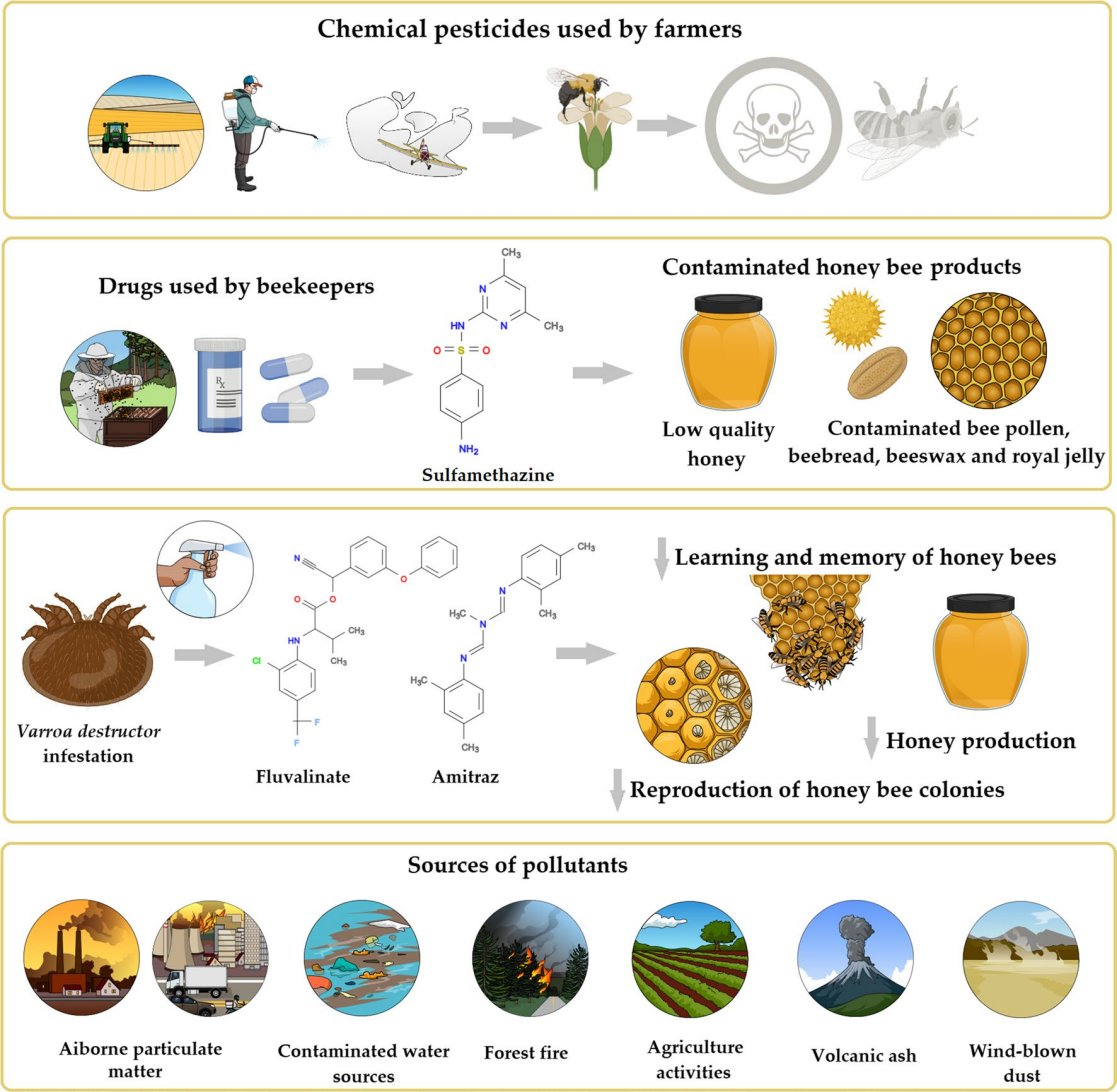
Different sources of contamination of bees and their products

Food control authorities have begun focusing on the problems related to harmful residues in beekeeping products as a result of consumers’ increasing awareness and also as a result of technological advances in this field (Karadas and Birinci 2018; Kasiotis et al. 2023; Le Conte et al. 2011). The use of chemicals in crop production is essential. The group of substances known as pesticides include insecticides, fungicides, herbicides, rodenticides, molluscicides, and nematicides. Researchers observed that less than 1% of the total amount of pesticides applied to control weeds and pests reaches the target pests. Depending on the type of pesticide used, it is a fact that a very large proportion of the chemicals used act as pollutants for the environment rather than being useful against the target organism (Gavrilescu 2005; Hernández et al. 2013). Furthermore, public concern regarding pesticide contamination of foods is increasing, due to residues from pollutants, dioxins, and resistance to pesticides. Nowadays, a variety of contaminants are present in areas where bee products are developed. For example, polycyclic aromatic hydrocarbons (PAHs) have toxic and carcinogenic effects and result from the incomplete combustion of organic compounds. According to reports, the high quantity of PAHs in bee bread and pollen, as well as the overly high suggested intake dose in these items, might seriously endanger human health if consumed on a regular basis (Al-Alam et al. 2019; Kasiotis et al. 2023). Pollution sources might be environmental (i.e., heavy metals, pesticides, bacteria, genetically modified organisms (GMOs), and radioactivity) or beekeeping-related (i.e., acaricides, bee repellents, pesticides, and antibiotics). An illustrative review of the most common contaminants in bee products can be found in Fig. 1.

There are a number of issues related to pesticide management, including pesticide regulation, a lack of resources for pesticide registration, safeguards against occupational pesticide exposure, consumer protection from food residues, and environmental protection from pesticide pollution (van den Berg et al. 2020). Given that some pesticides persist in nature for years, the health problems they can potentially cause become inevitable.

To date, one of the pressing challenges is the agricultural contamination with pesticides and antibiotics, which is rising due to continuous climate change and an increase in population size. This aspect is of particular interest as honey and other bee products are frequently used as food (in human nutrition) and medicine (as apitherapy). Pesticides, heavy metals, harmful microorganisms, and radioactive substances may contaminate honey and other bee products. Therefore, contamination of these products can pose serious health risks, not only to honey bees, but also to human beings (Yan et al. 2022). Antibiotic usage can result in an increase in antibiotic resistance in human or animal infections, while pesticide residues can disrupt cells and induce genetic modifications (Al-Waili et al. 2012; Jităreanu et al. 2022).

Due to its quality, fragrance, or health advantages, honey from various botanical and geographic sources presents a wide range in market value (Mărgăoan et al. 2021). Adulteration is frequently caused by the direct or indirect addition of less expensive sweeteners or low-quality honey.

Thus, it is essential to validate the geographic and botanical origins of the honey and identify the level of adulteration to safeguard consumer interests and the growth of the beekeeping business (Gün and Karaoğlu 2022; Ye et al. 2022; Zhang and Abdulla 2022). Melissopalynology is among the most widely implemented analytical methods for the characterization and/or authenticity determination of honey types (El-Sofany et al. 2020). Other analytical approaches may be employed based on their physico-chemical characteristics, including high-performance liquid chromatography (HPLC) and gas chromatography (GC), in order to identify specific markers and volatile compounds according to their botanical and geographical origins (Pita-Calvo and Vázquez 2018).

## Contaminants

### Chemical pesticides used by farmers

Pesticide usage is a common practice in agriculture, but its regulation needs to be applied in order to reduce the associated potential risks. The effects of these products have a negative impact on human health and the environment (Graham-Bryce et al. 1997; Möhring et al. 2020). According to Regulation No. 1107/2009 of the European Union (EU), the use of pesticides in the EU may not present an undesirable jeopardy or long-term effect on honey bees or have a detrimental impact on the survival and development of honey bee colonies. The regulation stipulates several stages to be completed before the approval of an active ingredient in a particular pesticide; in terms of honey bees, the most important steps are as follows: (1) One reporter Member State (MS) evaluates the provided data; (2) pesticide enterprises provide investigations on the effects of the active substance on honey bees and, if necessary, on residues in nectar and pollen; and (3) the European Food Safety Authority (EFSA) examines the active substance assessment in conjunction with MSs. Approved application rates and possible risk mitigation strategies are decided at the MS level, where products containing active substances are evaluated and authorized individually. Regarding the most used neonicotinoids, the EU regulations set maximum residue limits (MRLs) in honey for acetamiprid (0.05 mg/kg), clothianidin (0.05 mg/kg), imidacloprid (0.05 mg/kg), thiacloprid (0.2 mg/kg), and thiamethoxam (0.05 mg/kg). For certain acaridies, the MRLs in honey are as follows: acrinathrin (0.05 mg/kg), amitraz (0.2 mg/kg), cypermethrin (0.05 mg/kg), coumaphos (0.1 mg/kg), chlorfenvinphos (0.01 mg/kg), and fluvalinate (0.05 mg/kg). Conversely, there is a lack of MRLs for beeswax despite its significant use in pharmacological and food products (EU Commission 2023). Regarding the most extensively used fungicides, the MRLs in honey are as follows: azoxystrobin (0.05 mg/kg), boscalid (0.15 mg/kg), and up to 0.05 mg/kg for captan, cyproconazole, iprodione, and tebuconazole (EU Commission 2023).

For the chemical compounds used against pests, diseases, and weeds that lead to crop loss, 0.015 to 6.0% of the applied pesticides reach the target organism, obtaining the desired effect. The remaining 94–99.9% reaches non-target organisms and soil or merges with the surrounding natural ecosystems as chemical pollutants, as a result of drift and runoff (Graham-Bryce et al. 1997; Yıldız et al. 2013)*.* In addition, chemicals used against harmful organisms impair physiological development, including central nervous system function and reproduction. Research findings show that pesticides reduce the number of field bees in honey bee colonies, which are important for pollination, even when the dose is below the lethal amount (Hranitz et al. 2009; Schneider et al. 2012). In this regard, sublethal sulfoxaflor (0.3 ppb) exposure to honey bees impacts colony growth, foraging performances, and ultimately their production activity (i.e., reduced levels in honey, beebread, and brood) (El-Din et al. 2022). This is in accordance with other studies that revealed the negative impact of sulfoxaflor and flupyradifurone on honey bee survival rates and foraging activity. Chronic exposure to flupyradifurone alters both honey bees and bumblebees’ gut microbiota (Tamburini et al. 2021). Additionally, combined exposure to insecticides (sulfoxaflor and flupyradifurone) and fungicides (azoxystrobin) leads to gut microbiota dysbiosis, as evidenced by the relative abundance of *Serratia marcescens* (Al Naggar et al. 2022).

Regarding bee mortality, a total of fifteen insecticides were detected, including six naphthalene derivatives, three herbicides, one fungicide, one antiseptic/disinfectant, and one growth hormone, as a result of 16 suspected poisoning cases between 2006 and 2011 in Turkey (Ünal et al. 2010). A lower survival rate of forager bees leads to colony failure, irrespective of premature transitions of nurse bees to foragers. Furthermore, premature deaths in younger bees limit both brood development and forager replacement, which proves to be more detrimental compared to the statement presented above (Breda et al. 2022). In this aspect, over the course of 2 years, bees’ mortality and pesticide residues in bee bread have been evaluated. Acute bee mortality episodes occurred, particularly from April to June every year, due to increased accumulated levels of chlorpyrifos, dimethoate, and imidacloprid. Furthermore, beebread contained increased levels of the miticides amitraz (71.2 ng/g) and coumaphos (31.6 ng/g) (Calatayud-Vernich et al. 2019). The most common adverse effects reported in humans are altered sensorium, hyperglycemia, bradycardia, vomiting, and respiratory failure (Dhooria and Agarwal 2016; Ulukaya et al. 2001).

**Fig. 2:**
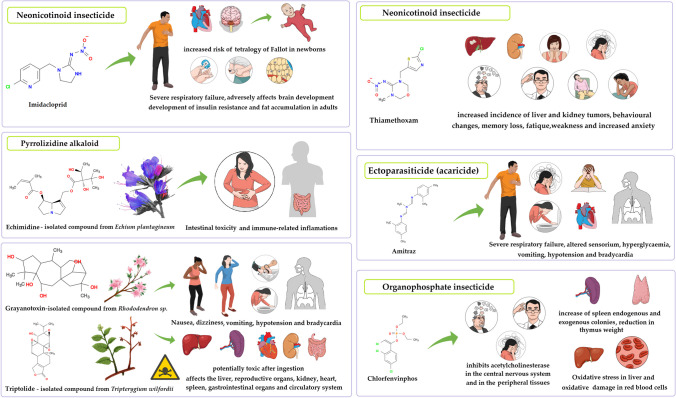
Mechanisms of toxicity in humans due to drug-active ingredient residues

Due to their effectiveness and safety, neonicotinoids are a class of pesticides that have undergone a considerable increase in use over the years. This group mainly includes imidacloprid, acetamiprid, clothianidin, nitenpyram, thiazine-derivatives, thiacloprid, and thiamethoxam. These compounds are particularly used in seed breeding and agricultural production to combat harmful pests. However, in recent years, the harmful effects of neonicotinoid pesticides on the health of bee colonies have caused increasing controversy and problems (Blacquière et al. 2012; Özdemir 2017). According to a trial’s findings, queen bees were contaminated with the typical neonicotinoid insecticides thiamethoxam and clothianidin while they were still under development. Neonicotinoids may negatively impact colony health by decreasing the frequency of queen bee mating. Furthermore, the colony’s survival is adversely affected by the worker bees’ decreased genetic diversity (Forfert et al. 2017). In a different aspect, using similar landscapes, covering seeds with an insecticide containing clothianidin and pyrethroid β-cyfluthrin on *Brassica* sp. oilseeds significantly decreased the number of wild bees (Rundlöf et al. 2015). Furthermore, corn and soybean seeds are also covered in neonicotinoid insecticides (i.e., clothianidin and thiamethoxam) as a protective layer (0.25─1.25 mg/seed) against pests. Regarding contact exposure, the amount of clothianidin (LD_50_) required for the death of a group of mature honey bees after 24 h ranges between 22 and 44 ng/bee, while for oral toxicity it is approximately 3 ng/bee (Environmental Protection 2003; Iwasa et al. 2004). In the case of thiamethoxam, the toxicity and LD_50_ are similar to clothianidin (Environmental Protection 2003). The concentrations of these compounds are sufficient to cause the death of an entire bee colony (Samson-Robert et al. 2014). It has been demonstrated that neonicotinoid compounds bind to nicotinic acetylcholine receptors, therefore affecting the honey bees’ brain (Tennekes and Sánchez-Bayo 2013). The antagonistic effect against these receptors in bees leads to paralysis and death, and the action depends on the selectivity of the insecticide (Fairbrother et al. 2014). According to research data, nitro-substituted chemicals (e.g., clothianidin, dinotefuran, and imidacloprid) and their metabolites, thiamethoxam and nitenpyram, are the neonicotinoid insecticides most harmful to bees. Despite the fact that imidacloprid and thiamethoxam had modest residual levels in bee bread and honey, exposure to these pollutants shows a significant effect on bee colonies (i.e., disrupts gut microbiota), as they prove to be more harmful to bees in both acute and chronic toxicity tests, particularly when combined with *Nosema cerenae* (Xiao et al. 2022). Imidacloprid especially decreases the abundance of *Lactobacillus*, *Serratia* sp., and *Snodgrassella alvi* in *Nosema*-infected honey bees (Balbuena et al. 2023). Furthermore, they also prove to be harmful to humans (Han et al. 2018), as they may negatively affect the development of the brain (Kimura-Kuroda et al. 2012) and induce tetralogy of Fallot and anencephaly in newborns (Carmichael et al. 2014; Yang et al. 2014), as well as heart failure and fat accumulation in adults (Park et al. 2013). *In vitro* studies revealed that imidacloprid increases the development of insulin resistance (Kim et al. 2013) (Fig. 2).

Thiamethoxam, another neonicotinoid insecticide, targets organs like the liver and kidney, and may eventually lead to cancer development (Rodrigues et al. 2010). Also, due to reduced acetylcholine levels, neurological symptoms may occur, such as behavioral changes, memory loss, fatigue, weakness, and increased anxiety in individuals (Marfo et al. 2015; Yi et al. 2023). On the other hand, cyano-substituted neonicotinoids (e.g., acetamiprid and thiacloprid) exhibit reduced toxicity toward bees (Decourtye and Devillers 2010), without disrupting the microbial community in the case of exposure to acetamiprid (Han et al. 2023) or combined exposure to fungicide penconazole and insecticide thiacloprid (Favaro et al. 2023). Conversely, other reports stipulate that thiacloprid residues in bee pollen exceeding 23 μg/kg lead to the complete loss of the bee colonies (0.00028 μg/bee) over the course of a 3-year study period (Beyer et al. 2018). Also, increased exposure of adult honey bees to thiacloprid (498 μg/kg) weakens their immunocompetence (Brandt et al. 2016). Even though the insecticides have been used according to EU legislation and national regulations, maximum residue levels of thiacloprid (1484 μg/kg) were detected in bee pollen collected from Finland apiaries (Kaila et al. 2022); however, these prove to be under the level of acute toxicity for honey bees. As of 2020, the EFSA banned the use of thiachloprid in open fields due to its negative impact on groundwater and human health. According to the EFSA, although sublethal or synergistic effects exist, the risks to honey bee health are low (European Food Safety et al. 2019). Future research investigations must be carried out considering other factors that are relevant to the field, such as concentrations, exposures, and evaluation times. By gaining a better understanding of neonicotinoids’ mechanism of action in bees (generally through their interaction with specific receptors), molecular markers may be used to increase risk assessment and to identify or develop potential environmentally-friendly compounds (Blacquière et al. 2012; Thompson et al. 2020).

Other classes of frequently used pesticides are represented by organophosphorus and pyrethroid insecticides, which are known for inducing myocardial and neuromuscular impairment in humans, as well as acute toxicity for honey bees (Wang et al. 2020; Yao et al. 2020). According to research carried out in and around Isparta Province in Turkey, residual amounts of pesticide residues were detected in seven filtered flower honey samples, including diazinon, chlorpyrifos, malathion, ethion, cypermethrin, and deltamethrin (Canbay et al. 2012; Chauzat et al. 2009).

During a period of 4 years, a study of 64 possible pesticide residues in pollen, nectar, and beehive components from several honey production locations in China was conducted. According to the findings, method detection limits (MDLs) reached or exceeded their levels in 96.6% of bee bread, 93.6% of pollen, 81.5% of nectar, and 49.3% of honey that contained a minimum of one pesticide. A number of 19 pesticides were detected; the most frequent one was carbendazim, found in almost all samples (>85%) (Xiao et al. 2022).

A 3-year survey on pesticide residue levels detected in bee collected pollen (BCP) from different Italian regions revealed the presence of several insecticides and fungicides with exceeding ADI (Acceptable Daily Intake) and MRL (Maximum Residue Limit) levels. The insecticides chlorpyrifos, dimethoate, and phenthoate exceeded ADI and MRL levels over the years. Overall, chlorpyrifos was detected in 30% of all samples, with the highest level of 46% in 2014. In the case of fungicides, metalaxyl presented the highest residue level (60 μg/g) in the region of Veneto (Giavera del Montello) exceeding the MRL levels. Pesticide contamination persisted in several apiaries all year-round and throughout time, for instance at Cisterna d'Asti (Piemonte) and Ponte in Valtellina (Lombardia) (Tosi et al. 2018). To determine the relevance of pesticide exposure in the field assessment of unexplained honey bee colonies, 330 colonies from Belgium were evaluated from July 2011 to May 2012. Samples of honey, bees, beeswax, and bee bread were collected and contained 99 pesticides, including five common viruses and 41 fungicides, 39 insecticides and synergists, 14 herbicides, 5 acaricides, and metabolites. The majority of residues were found in beeswax (Simon-Delso et al. 2014). Other frequently used herbicides, such as boscalid and iprodione, may reduce the growth and development of larvae. Furthermore, they might induce mitochondrial damage, inflammation, and DNA damage in humans, due to their highly detected levels in wax and bee bread (d’Hose et al. 2021; Simon-Delso et al. 2014; Washington and Tchounwou 2004). The exceeding limits detected in these studies revealed that environmental pesticide contamination levels are concerning for the health of humans, bees, and the ecosystem. Furthermore, BCP monitoring is an effective method for detecting unauthorized pesticide usage as well as environmental pesticide pollution.

When it comes to fungicide usage, it has been observed that honey bees are chronically exposed to different doses of such chemicals. In general, this class of pesticides impacts both larval and adult stages of development. Moreover, the co-occurrence of fungicides with other types of agrochemicals seems to act synergistically in raising the toxicity towards bees. Exposure to sub-lethal doses of penconazole alters the foraging preferences of honey bees for *Hedera* pollen (Favaro et al. 2023).

Samples of bee bread were collected from colonies pollinating orchards in seven different sites over a 2-year period and further examined for fungal composition and fungicide residues. The presence of cultivated orchards within the flying range of honey bees may be the reason why an organic orchard contains the highest concentration and diversity of fungicides (Rondeau and Raine 2022). Despite the differences in their presence in the environment, *Penicillium*, *Aspergillus*, *Rhizopus*, and *Cladosporium* (beneficial fungi) have been identified as the most common fungal isolates. Even within the same apiary, some differences in the fungal components were observed between colonies. Several variable elements have been identified, including *Absidia*, *Alternaria*, *Aureobasidium*, *Bipolaris*, *Fusarium*, *Geotrichum*, *Mucor*, *Nigrospora*, *Paecilomyces*, *Scopulariopsis*, and *Trichoderma*. As a result of fungicide contamination, fewer fungal isolates were found. Increased fungicide levels have been observed to have a specific impact on *Aspergillus* abundance (Yoder et al. 2013).

Traces of pesticides found in honey are important not only for consumers’ knowledge, but also due to exceeding pesticide levels in honey bees that may pose a threat to their health. Recently, the losses in bee populations in some countries have drawn increasing attention, as colony performances and honey bee activities may be affected due to exposure to varied chemicals (Canbay et al. 2012). A reviewed list of pesticides used in different countries, as well as their negative impact on honey bees, can be visualized in Table 1.

**Table 1:** Active substances used as pesticides, detected in bees and bee products in some countries

Country	Active substances	Product or organism	Negative effects observed	References
Brazil	Azadirachtin, chlorantraniliprole, imidacloprid	Honey bee (*Apis mellifera*, *Megachile rotundata*, *Nomia melanderi*)	Induced mortality, delayed development, reduced number of larvae, hyperactivity, and trembling ↑ acute toxicity of imidacloprid (60 ng/bee at 48 h and 40 ng/bee at 72 and 96 h of exposure) ↑ mortality (>90%) of worker bees after oral exposure to imidacloprid (1.0x), and ↔ mortality to azadirachtin (40%) and chlorantraniliprole (>30%)	Naiara Gomes et al. 2020
Belgium	Boscalid, captan, coumaphos, iprodione, pyrimethanil	Beeswax, bee bread, honey, worker bees, queen bee	Low acute toxicity to bees, growth, and development of larvae; ↑ residue levels in bee bread and beeswax (0.21–3.1 mg/kg) compared with honey (0.001–0.058 mg/kg) ↑ boscalid levels (58.4 mg/L) detected in nurse bees and bee bread (0.005−1.3 mg/kg) ↑ captan (3.1−1.9 mg/kg) and iprodione (0.24−1.5 mg/kg) levels in bee bread and beeswax	DeGrandi-Hoffman et al. 2013 and Simon-Delso et al. 2014
Australia	*Tau*-fluvalinate	Honey bee	↓ colony productivity; no adverse effects on brood and bee numbers and food storage	Colin et al. 2021
USA	Fluvalinate, coumaphos, chlorothalonil, chlorpyrifos	Pollen, beeswax	↑ larval toxicity with chlorothalonil (34 mg/L) and fluvalinate (3.0 mg/L) combination (56−68% mortality) of 6 day old larvae; ↓ toxicity of fluvaliante and chlorothalonil mixture when combined with coumaphos ↑ sensitivity of 4−5-day-old larvae (32% mortality) to chlorpyrifos (1.5 mg/L)	Zhu et al. 2014
	Amitraz, coumaphos, *tau*-fluvalinate	Queen bee	↓ nursing visits of queen bee in the amitraz group compared to the control group ↑ ovarioles per queen ovary in coumaphos and *tau*-fluvalinate group	Walsh et al. 2021
Italy	Chlorpyrifos, imidacloprid	Bee pollen	Exceeding MRL of chlorpyrifos (179 μg/kg) in 30% of the samples, and ↑ hazard quotient level (HA=5054) of imidacloprid (19−17 μg/kg) in 12% of samples and 50% bee mortality after 10 days; exceeding Acceptable Daily Intake (ADI) levels in bee pollen samples (*n*=166) with chlorpyrifos (34−74 μg/kg)	Tosi et al. 2018
Turkey	Diazinon, chlorpyrifos, malathion, ethion, cypermethrin, deltamethrin	Honey	Residue levels between 0.5 and 3.5 ng/g; ↓ accumulation levels of pesticides	Canbay et al. 2012
	Thiacloprid	Honey bee (*A. mellifera anatolica*, *A. mellifera causica*)	↑ mortality rate in both Anatolian (96.7%) and Caucasian (96.5%) honeybees with increased thiachloprid concentration (0.4 mL)	Karahan et al. 2018
USA	Fungicide	Bee bread, *A. mellifera*	Colonies positive for chalkbrood, *Aspergillus*, and *Penicillium* isolates from bee bread	Yoder et al. 2013
Czech Republic	Fungicides (azoxystrobin, boscalid, cyproconazole, tebuconazole) Insecticides (chlorpyrifos, *tau*-fluvalinate, thiacloprid)	Bee bread	High levels in fungicides (azoxystrobin, 0.01−0.03 mg/kg; boscalid, 0.002−0.04 mg/kg) and insecticides (chlorpyrifos, 0.004−0.02 mg/kg; thiacloprid, 0.004−0.11 mg/kg)	Bokšová et al. 2021
China, France	thiamethoxam	Honey bee	↓ survival, emergence and physiology with increased thiamethoxan concentrations (1.44 ng/ mL) ↑ level of concern in nurse and worker bees with exposure to thiamethoxan (16.7 ppb)	Tavares et al. 2017 and Wang et al. 2022
Egypt	Imidacloprid, thyamethoxan, dinotefuran	Honey bee (*A. mellifera*), bee pollen, honey	↑ levels in imidacloprid desnitro –HCl (2.4−26 ng/g) in both spring and summer honey bees, imidacloprid−5−hydroxy in honey bees (2.1–41.6 ng/g) and bee pollen (1.0–34.2 ng/g); dinotefuran in bee pollen (1.2–17.4 ng/g) and honey bees (0.3–74.3 ng/g); thiamethoxan in honey (18.8 ng/g);	Codling et al. 2018
Canada	Organophosphorus insecticides	Honey bees (*A. mellifera*), beebread, honey	Diazinon (0.3 ng/g), dimethoat (1.5 ng/g), and chlorphyrifos-oxon (0.2 ng/g) detected in honey; chlorpyrifos (2.7 ng/g), chlorpyrifosmethyl (15.8 ng/g) and fenamiphos (0.4 ng/g) detected in beebread; dichlorvos (889.2 ng/g), malathion (3.7 ng/g), and ethoprop (1.4 ng/g)	Al Naggar et al. 2015b
Canada	Clothianidin, imidacloprid, thiamethoxam	Honey bee (*Apis mellifera*), and hive matrices (bee pollen, honey)	↑ levels in clothianidin in honey bees (52%), pollen (57%), and honey (67%); increased levels in imidacloprid in honey (52%) and pollen (5%); high levels in thiamethoxam in honey (75%) and pollen (21%)	Codling et al. 2016
Indonesia	Imidacloprid, deltamethrin	Honey, *A. mellifera*, *Tetragonula laeviceps* (stingless bee)	None, lower levels of insecticides; LOD (<0.001 mg/kg imidacloprid and <0.003 mg/kg deltamethrin); MRL (0.05 mg/kg imidacloprid and 0.03 mg/kg deltamethrin)	Mubin et al. 2023

Note: *ND* not detected, *LOD* limit of detection, *MRL* maximum residue limit

### Drugs used by beekeepers

One of the problems of beekeeping is related to the existence of a variety of pathogens that can be present in bee colonies. Even though to some extent honey bees are able to provide protection against pathogens, this resistance can change according to varied factors. An illustrative and common example is the introduction of pathogens due to extensive global trade. Consequently, the natural defense potential of honey bees needs to be enhanced through the addition of different antimicrobial drugs, such as antibiotics and acaricides. Among the diseases that can be cured using such pharmaceuticals are as follows: American and European foulbrood, *Nosema* infection, wax moth infestations, and tracheal mites (Nagaraja and Rajagopal 2019; Ortiz-Alvarado et al. 2020).

#### Antibiotics

Beekeepers frequently use antibiotics to eradicate illnesses in honey bees (Yıbar and Soyutemiz 2013). However, the quality of honey may be lowered by the presence of xenobiotics and be detrimental to people’s health. Moreover, the widespread use of antibiotics has the potential to develop strains of bacteria that are resistant to a variety of medications, with severe therapeutic implications (Bargańska et al. 2011).

In a study conducted in Turkey, residues of some antibiotics such as sulfadimidine, tetracycline, and streptomycin were found in several honey samples, although their use in beekeeping is not legally permitted. Tetracycline group antibiotics were frequently detected in honey, and residues of both antibiotics were determined together in some samples. This suggests that some drugs used in beekeeping may have two active ingredients, both sulfa- and tetra-antibiotics (Sunay 2006). In another study, it was reported that the residues did not pose a risk to food safety in most samples, although residue levels above the MRL were detected in honey samples. Out of the total 210 samples, multiple antibiotics have been detected, mainly 59 for sulfamethazine (29.5%), 7 for tetracycline (3.5%), and 22 samples (11%) for streptomycin. Regarding pesticides, 13 samples (6.5%) tested positive for amitraz and 3 for coumaphos (1.5%). In a positive aspect, all samples were fluvalinate residue-free, highlighting the fact that beekeepers excluded fluvalinate as a treatment against mites. However, in 52.5% of the honey samples obtained from the various areas, no traces of veterinary medication residues were found (Gül and Sahinler 2017).

Streptomycin residues in honey from individual apiaries and retail marketplaces in six different districts of Kosovo were assessed in 2017. Streptomycin residues were detected in 34 (25.9%) out of 131 honey samples and in a significant number of imported honey samples in 11 (45.8%) out of 24 samples, respectively. According to published reports, none of the positive tests exceeded the upper limits for residue established by European Union (EU) standards. Regarding honey bee development during antibiotic usage, it has been revealed that the timing of antibiotic treatment not only has an effect on lipid levels, but also on behavioral development. Thus, during the larva-pupa stages, an accelerated development was noticed compared with the larva-adult stages, which showed a delayed development. A loss of abdominal lipid stores was noticed in both developmental stages with similar results among the tested antibiotics (i.e., oxytetracycline and tylosin tartrate) (Ortiz-Alvarado et al. 2020). Antibiotic treatments have also been linked with the alternation of gut microbiota in honey bees, which leads to sensitivity to viral and/or bacterial infections (Deng et al. 2022; Raymann et al. 2017) and eventually a reduction in their survival rates (Li et al. 2019). Thus, treatment with tetracycline promotes Israeli acute paralysis virus (IAPV) susceptibility and, at the same time, it disrupts the abundance of *Lactobacillus* in the gut of *Apis cerana*, known for its prominent antiviral role (Deng et al. 2022).

The treatment using tetracycline at higher concentrations (LC_50_ = 125.25 μg/ml) leads to the loss of half of the individuals (Aljedani 2022). Furthermore, streptomycin-exposed bumblebees exhibit decreased learning and foraging activities (Avila et al. 2022). Although beekeepers expect losses every year, with a rate of 16% being considered acceptable, an increased rate of antibiotic treatment leads to a colony reduction of up to 40% (Kulhanek et al. 2017). Simulation experiments suggest that if the antibiotic treatment causes dysbiosis-induced mortality for 60–365 days or 120–365 days per year, then the target of reducing colony size by 7% and 20%, respectively, would be exceeded after only 1 year (Bulson et al. 2021). Furthermore, when exposed to antibiotics, honey bees become susceptible to bacterial infections, such as *Serratia marcescens.* This bacteria increases mortality rates in both larval and adult stages of honey bees exposed to antibiotics. In this aspect, Motta and his collaborators discovered that caffeine (in 1 mM concentration), which is an important stimulant for humans, alleviates the negative effects and fights against bacterial infections in honey bees, particularly those caused by *Serratia marcescens* (Motta Erick et al. 2023). Further studies should assess the roles of different plant-derived metabolites that alleviate antibiotic-caused infections in honey bees.

#### Acaricides

Amitraz and fluvalinate are highly effective acaricides used against *Varroa destructor* infestations in honey bee colonies. Although honey bees are more resistant to acaricides, these chemicals have adverse effects on honey bees’ reproduction, olfaction, and honey production (Lim et al. 2020). Thus, it was determined that amitraz and fluvalinate damage honey production and the reproduction of honey bee colonies. In addition, fluvalinate causes a decrease in the olfactory senses of honey bees, reduces honey productivity, and affects the learning and memory of honey bees (Ilyasov et al. 2021). Details regarding the presence of acaricides in bee products can be visualized in Table 2.

**Table 2:** Several bee drug-active ingredient residues in bee products

Country	Active substances	Bee product	References
Russia, Korea	Amitraz (20 μg/bee) and fluvalinate (2 μg/bee)	↓ oviposition of queen bees compared to control ↓ honey production (24–27 kg) compared to control (31 kg) ↓ expression of olfactory-related neuropeptide genes in fluvalinate-treated group	Ilyasov et al. 2021
Belgium	Fungicides (boscalid, captan, iprodione)	↑ levels of boscalid residues in beeswax (0.29 mg/kg), and bee bread (0.4−1.3 mg/kg) ↑ levels of captan in beeswax (3.1 mg/kg) and bee bread (1.9 mg/kg) ↑ iprodione levels in beeswax (0.2–1.5 mg/kg) and bee bread (0.3–1.5 mg/kg)	Simon-Delso et al. 2014
Switzerland	Chloramphenicol	Honey (0.4−6.0 μg/kg)	Ortelli et al. 2004
	Coumaphos and *tau*-fluvalinate	↑ Residue levels of coumaphos (401 μg/kg) and *tau*-fluvalinate (236 μg/kg) in beeswax	Marti et al. 2022
Turkey	Naphthalene	↑ levels in honeycomb honey (3.0–8.9 μg/kg)	Çakar and Gürel 2019
	Tetracycline, streptomycin	↑ tetracycline (1.7–13.9 ppb) and streptomycin (8.2–25.8 ppb) levels in liquid honey	Ağaoğlu et al. 2020
India	Oxytetracycline, erythromycin	↑ oxytetracycline (28.9 ng/g; 15% above MRLs) and erythromycin (78.8 ng/g; 5% above MRLs) residue levels in honey	Kumar et al. 2020
Greece	Tetracycline	↑ tetracycline levels (0.01–0.39) in honey samples originated from the Thrace area	Saridaki-Papakonstadinou et al. 2006
	Coumaphos (Perizin and Checkmite+)	↑ residue levels of coumaphos (0.58−12.52 mg/kg) in royal jelly (natural queen cells) after acaricide application	Karazafiris et al. 2022
Romania	Carbendazim, enilconazole, tebuconazole, thiabendezol	↑ enilconazole levels (3.2–4.1 μg/kg) in almost all honey samples ↑ carbendazim levels (5.3–5.4 μg/kg) in sunflower honey ↑ tebuconazole levels (2.9–3.1 μg/kg) in spring, rapeseed, and linden honey (Iași) thiabendazole (3.3 μg/kg) detected in linden honey (Iași) buckwheat (Sibiu) and polyfloral (Vâlcea) honey were free of residues	Blaga et al. 2020
Hungary	Coumaphos, fluvalinate, acrinathrin	↑ coumaphos levels (4−374 ng/g) were detected in 89% of bee pollen samples ↑ fluvalinate levels (2−72 ng/g) detected in 47% of bee pollen samples ↑ acrinathrin levels (1−458 ng/g) in 20% of bee pollen samples	Calatayud-Vernich et al. 2018
Spain	Chlorfenvinphos, cypermethrin, acrinathrin	↑ amitraz (52%), coumaphos (33%), acrinathrin (28%), and chlorfenvinphos (11%) levels in beeswax	Albero et al. 2023

To determine the incidence of beeswax pesticide residue in Spain, 35 samples were collected in 2016. Beeswax is equally contaminated with acaricides and, to a much lesser extent, with pesticides and fungicides. Miticides applied inside the hive contributed to more than 95% of the average pesticide load. Commonly used acaricide compounds such as coumaphos (100%), fluvalinate (86%), and amitraz (83%) were the most frequently detected pesticides. Pesticide assessment in beeswax can be a tool for monitoring veterinary treatments by beekeepers and the exposure of honey bees to environmental pollutants (Calatayud-Vernich et al. 2017). The effects of Apivar® and Thymovar® as fall treatments against *Varroa* *destructor* have been evaluated by monitoring both the winter survival rate and viral loads. The colony infestation rates drastically decreased in the Apivar®-treated groups compared with both the control and Thymovar®-treated groups. An increased efficacy of Apivar® (76%) against *V. desctructor* after 22 days compared with Thymovar® (26%) has been observed. Additionally, reduced Apivar concentrations in bees (15 ng/g) have been recorded, as compared with significantly higher concentrations of thymol (64.800 ng/g) (Al Naggar et al. 2015a).

Although amitraz has the ability to eradicate mites in honey bee colonies, its metabolites and residues are contaminants in honey products. According to one study, after colonies received different Apivar® doses, the residual levels of amitraz and its metabolites in honey and beeswax changed. Amitraz metabolites, specifically 2,4-dimethyl-phenyl-formamide (DMPF) and 2,4-dimethyl-aniline (DMA), were found in samples after 28 days of treatment; however, amitraz residues were not found in honey and beeswax after 42 days of being administered in colonies in the experiment. According to reports, DMPF residue levels in honey and beeswax samples do not exceed the maximum residue limits (MRLs), which range from 13.7 to 60.5 μg/kg and 196 to 6.160 μg/kg, respectively. The dosage used increased the possibility of determining residue levels (Chaimanee et al. 2022). In Greece, the contamination level in royal jelly produced from colonies chemically treated with coumaphos (CheckMite + and Perizin) and *tau*-fluvalinate (Apistan) using synthetic plastic queen cells was assessed 42 days following the application. Utilization of CheckMite + strips during manufacture has been reported to result in higher levels of acaricide residues in the final product as opposed to Perizin and Apistan residues. Therefore, CheckMite + strips were also applied to assess product contamination after a long period. Findings showed that contamination of royal jelly generated in plastic queen cells declined with time, and 1 month after strip removal, no residue had been detected. On the contrary, the contamination of royal jelly collected from natural queen cells proved to be higher. Thus, even in low quantities, coumaphos may be transported from beeswax to royal jelly (Karazafiris et al. 2022).

In Italy, acaricide residues in beeswax are a common occurrence. In a 10-year survey of residues, 50% of the samples revealed the presence of coumaphos, followed by fluvalinate (38%) and chlorfenvinphos (25%) (Boi et al. 2016). Surprisingly, even if chemically synthesized products are not allowed in organic system productions, coumaphos has been detected in a small percentage of organic honey in Italy (Chiesa et al. 2016). In some cases, honey samples in Italy exceeded the maximum residue limits established by the European Community for chlorfenvinphos, coumaphos, and *tau*-fluvalinate (Saitta et al. 2017). Out of these, although it is forbidden for use against *Varroa*, chlorfenvinphos was present in high levels in all beeswax samples collected in June and December from several apiaries in Andalusia, Spain (Albero et al. 2023). Chlorfenvinphos is known to induce oxidative damage in liver and red blood cells and antioxidative enzymes, with an increase in malondialdehyde levels (Łukaszewicz-Hussain et al. 2008; Sosnowska et al. 2013b). Furthermore, strong correlations have been observed between acetylcholinesterase inhibition and behavioral impairments (Raszewski and Filip 2009; Sosnowska et al. 2013a).

Extensive research was conducted on pesticide residues in bee colonies, BCP, and bee wax. Multiple residues prevailed in the honey bee, BCP, and wax samples, with 2 or more pesticides detected in 92.3% of the 749 analyzed samples. With regard to BCP and wax samples, the highly detected combined pesticides were fluvalinate and coumaphos in 83% of the samples, followed by fluvalinate with chlorothalonil in 50% of the samples. The highest concentrations of fluvalinate (7329.5 ppb) and amitraz (1080.7 ppb) were detected in wax, whereas in BCP high levels of chlorothalonil (1593.5 ppb) were detected (Mullin et al. 2010). An average of seven chemicals per BCP sample were detected; therefore, it seems likely that various pesticide interactions could have a negative impact on the health of honey bees. The fungicide chlorothalonil detected in the highest amount in BCP proves to be a marker for “entombing” behavior in honey bee colonies, which acts as a defensive behavior for contaminated foods stored in the hive (vanEngelsdorp et al. 2009a). Acute and sublethal declines in honey bee fitness, particularly in queen bees, are caused by prolonged exposure to high concentrations of these persistent neurotoxicants (Collins et al. 2004). Conversely, higher coumaphos levels prove to be beneficial to the hive as they have been associated with mite control (VanEngelsdorp et al. 2009b).

#### Other drugs

The contact with pesticides, climate change, and the reduction of flower diversity (i.e., reduced protein amounts due to a lack of pollen sources) lead to several consequences, such as mass mortality in bees, colony collapse disorder, and even reduced resistance of worker bees to parasites (Aufauvre et al. 2012; Goulson et al. 2015; Huang 2012; Matsumoto 2013; Yalcin et al. 2021). In this regard, another significant type of infection in bees is represented by nosemosis, caused by the microsporidia *Nosema ceranae*. This parasite can induce bee colony mortality, the reason why sometimes the administration of a treatment is necessary (Marín-García et al. 2022). Fumagillin is an example of a chemical compound used against nosemosis, both as a control and as a prophylactic. Fumagillin not only affects honey bees’ physiology by altering midgut proteins (Huang et al. 2013), but also poses a risk to human health due to its presence as residues in honey (Nozal et al. 2008; van den Heever et al. 2015). Alternative approaches, such as plant extracts, isolated compounds from natural products and/or essential oils (EO) are important, since fumagillin is the sole approved chemical treatment against nosemosis (El-Seedi et al. 2022). *Cryptocarya alba* essential oil and *Olea europea* extract proved to be the most effective in a dose-dependent manner. Thus, 4 μg of *C. alba* essential oil/honey bee exhibited 80% spore inhibition, closely similar to the fumagillin-treated group (Bravo et al. 2017), whereas 10 mg/mL of *O. europea* extract exhibited 99% spore inhibition on the 7th day (Arenas 2022). Bee venom is recognized for its significant antimicrobial activity; however, few studies have investigated its potential use against *N. ceranae*. In this regard, the administration of sugar syrup containing bee venom at both LC_10_ and LC_20_ significantly reduces the fungal pathogen infection and increases bee survival rates, particularly on day 12 of the treatment. The effects are also associated with elevated levels of nemocyte count and expression of AMP-encoding genes. Therefore, plant extracts and bee venom might provide non-antibiotic options for *Nosema* management and contribute to minimizing antibiotic usage (Chaimanee et al. 2021; Mahmoud et al. 2024).

The use of probiotics, particularly *Bifidobacterium* sp. and *Lactobacillus* sp., is another effective treatment against *Nosema* infection. Infected bees fed with sugar syrup mixed with lactobacilli and bifidobacteria led to a 90% reduction in *N. ceranae* load and a 47% reduction in infected bees (Baffoni et al. 2016).

A total of 60 honeycomb and filtered honey samples gathered from beekeepers in Antalya province were tested for the presence of pesticides, antibiotic components, and naphthalene residues. The research revealed that only three comb samples contained naphthalene residue levels between 3.0 and 8.9 μg/kg, which is below the 10 μg/kg level specified for naphthalene in the Turkish Food Codex Honey Regulation (Çakar and Gürel 2019; FAOLEX Food and Nutrition n.d.).

The greater (*Galleria mellonella* L.) and lesser (*Achroia grisella* Fabricius) wax moths are other pests that can cause significant damage to bee colonies, through the destruction induced by caterpillars. The incidence of this pest has to be reduced through several methods, including physical and chemical ones. Ultimately, the usage of natural products that have been proven to be harmless to honey bees but effective against pests could reduce the risk of toxicity, acting as a reasonable alternative (Ellis et al. 2013; Telles et al. 2020).

### Environmental pollutants

Bees’ preferred food source has a significant impact on the composition of honey, as well as on its therapeutic benefits. Consumers and the pharmaceutical industry both use various types of honey, each with unique properties. Honey products are utilized in face creams, toothpaste, and other dermato-cosmetic and hygiene products. Furthermore, useful components, such as honey and propolis, are found in tinctures, syrups, suspensions, and other therapeutic products for internal use. In locations where mining, manufacturing, and agriculture are practiced, heavy metal toxicity is a hazard to the environment. Heavy metals in higher concentrations are lethal for honey bees; moreover, the residue in bee products represents a threat to human health (Burden et al. 2019; Mititelu et al. 2022; Topal et al. 2022).

Consumers’ usage of high-quality honey is essential; on the other hand, honey is one of the most frequently adulterated products. However, the level of contamination with different harmful chemicals in the environment is significantly influenced by its quality. Various research studies have been conducted to explore the chemical transfer from soil to plants and ultimately to bees’ organisms and their products (Goretti et al. 2020; Tomczyk et al. 2020). In order to prevent contamination, installing beehives close to polluting businesses or in regions where they are being created must be avoided; this is especially important in the case of industrial settings that are highly polluted with heavy metals. Breed selection, growth management techniques, best-quality feed, and proper housing in sanitary circumstances are the cornerstones of disease prevention in organic beekeeping. Allopathic veterinary medications and those produced through chemical synthesis are prohibited for the treatment of bees (FAO Food and Agriculture Organization of the United Nations 2018; Mititelu et al. 2022).

The key to the determination of ecotoxicological risk related to an animal is whether it can actively reject foods contaminated with dangerous compounds through taste. During lab tests, bees were administered food poisoned with arsenic, lead, or zinc without showing any preference at estimated levels of 929.10 μg/g As, 6.45 mg/g Pb, and 72.46 mg/g Zn. Appetitive responses indicating reduced intake and metal detection have been reported to be observed only at the highest concentrations of zinc (122.3 mM) and lead (3.6 mM) by the interaction between the antenna and proboscis. Overall, cellular and behavioral responses have been reported to provide no evidence suggesting the existence of particular mechanisms that enable the selective detection of harmful metals (such as arsenic and lead) as opposed to zinc, which is essential for many biological processes. This evidence suggests that honey bees are only capable of avoiding metal contaminants in their diet at extremely high concentrations, which are very unlikely to occur in the natural world. It was discovered, however, that honey bees are unable to recognize the moderate yet dangerous amounts of contaminants present in flowers, which poses a serious concern (Monchanin et al. 2022).

The average amounts of iron (158516 μg/kg), copper (7104 μg/kg), zinc (29482 μg/kg), lead (899 μg/kg), and cadmium (34 μg/kg) in BCP samples were found to be higher than the World Health Organization’s (WHO) permissible limits, according to research comparing the heavy metal levels in several bee products originating in the Turkish province of Aydın. Among chemical elements, barium, beryllium, chromium, cobalt, iron, lead, lithium, nickel, tellurium, uranium, and zinc showed the highest concentration in propolis samples from all the examined bee products; on the other hand, boron, copper, manganese, and rubidium showed the highest accumulation in BCP samples (Bakırcı 2019). In BCP samples collected from three distinct regions (alpine, plain, and close to an urban zone) in Ukraine, heavy metals have been found to be present at greater concentrations in urban areas (Klym and Stadnytska 2019). Furthermore, BCP samples collected from six colonies located in İzmir Province (Turkey) presented a high amount of arsenic and lead, which were significantly high during the summer months and in September (Topal et al. 2022). In a different study, honey samples collected from industrialized areas accumulated higher levels of Zn (3.99–3.09 mg/kg) and Cu (2.54–1.03 mg/kg), with the lowest level in Cd (0.07–0.02 mg/kg) (Mititelu et al. 2022).

Prior to employing bee products as dietary supplements, their quality should be evaluated for the presence of hazardous heavy metals. Since bee products are extremely heterogeneous and their elemental content varies depending on the environment, regulations defining acceptable inorganic pollutant levels should be established (Matuszewska et al. 2021).

Industrial processes, including mining and processing metal ores and coal, extracting phosphate for fertilizer production, hydraulic fracturing, and heavy irrigation or fertilizing agricultural land are major causes of metal and metalloid contamination (Järup 2003; Li et al. 2014a). In terms of honey bees’ health, contaminated water has been overlooked as an exposure channel and as a means of transportation. (Perugini et al. 2011; Porrini et al. 2003). Moreover, honey bees prove to be good bioindicators of environmental pollution (Costa et al. 2019; Traynor et al. 2021). Contaminated waters, whether from contact or ingestion, might be harmful to honey bees if they contain trace amounts of pesticides.

As a persistent pollutant, airborne particulate matter (PM) can originate from both natural and artificial sources. Natural sources include sea salt, volcanic ash, wind-blown dust, soil particles, pollen, leftovers from forest fires, and the oxidation of biogenic reactive gases (Kelly and Fussell 2012; Kim et al. 2015). Anthropogenic factors include burning fossil fuels (such as in vehicles and power plants), eroding pavement from vehicle traffic, abrasion from brakes and tires, and industrial operations (i.e., metals, ceramics, and brick manufacturing), as well as construction, smelting, quarrying, and agricultural activities (Kelly and Fussell 2012; Kim et al. 2015; World Health 2013).

PM may be divided into three categories per aerodynamic diameter: coarse PM_10_, fine PM_2.5_, and ultrafine PM_0.1_ (Brook et al. 2010; Juda-Rezler et al. 2011; Kelly and Fussell 2012). PM < 1 μm may remain airborne for days or weeks, making them susceptible to long-range transboundary air movement (Juda-Rezler et al. 2011; World Health 2013). In worker bees, the abundant pubescence enhances the accumulation of an electrical charge that attracts small airborne particles (Bonmatin et al. 2015; Negri et al. 2015; Vaknin et al. 2000), and multiple studies have been conducted on the contamination of bees by PM located in urban, periurban, industrial, and mining areas (Capitani et al. 2021; Negri et al. 2015; Papa et al. 2021a; Pellecchia and Negri 2018). On the other hand, research on the contamination of bee products by airborne PM is still scarce. For instance, metal-based particles from automotive traffic contaminate BCP and honey in metropolitan settings (e.g., Fe-based compounds, metallic Zn, barite, and antimony oxide) (Papa et al. 2021a).

Research exploring the effects of oral exposure to pollutant PM on bees focused on alterations in the gut microbial population or cytological and histological abnormalities of the gut epithelium (Al Naggar et al. 2021). Worker bees exposed to submicrometric TiO_2_ particles, a widespread airborne contaminant, showed differences in the bacterial community and alterations in the abundance of putative probiotic species (Papa et al. 2021b).

PM pollutants also include microplastics (MPs) and nanoplastics (NPs). MPs are particles with sizes ranging from 5 to 1 μm, whereas NPs have sizes smaller than 1 μm (Allen et al. 2019). MPs and NPs are pervasive, man-made particles that are easily suspended in the atmosphere due to their low density. Erosion of synthetic rubber, personal care products and microbeads in cosmetics, plastic shot used in industrial abrasives, granules of resin used in the production of plastics, synthetic textiles from soft furnishings and clothing, and city dust are regarded as one of the most significant sources of primary MPs transferred into the ocean (Abbasi et al. 2019; Prata 2018). In 12% of honey samples recently collected in Ecuador, MPs were reportedly found. Moreover, MPs have been discovered in honey bees gathered from Danish beehives and from surrounding semi-urban and rural locations. After being exposed to polystyrene (PS)-MPs, honeybees experienced alterations related to oxidative damage, detoxification, and immune-related gene expression, which together decreased the richness of their gut microbiota (Al Naggar et al. 2021).

Honey and propolis were gathered from beekeepers with Romanian accreditation who set up hives in two regions with various industrial activities. Area 2 (A2) was virtually free of industrial activity but was classified as having moderate agricultural activity. Area 1 (A1) was a region of high industrial activity, with additional enterprises nearby, including a refinery. A total of 144 samples were collected, 12 for each kind of honey, propolis, and soil. The study’s findings draw attention to the possibility of contamination with different pollutants originating from the soil or other sources, as well as the accumulation of these harmful pollutants in concentrations that could be detrimental to consumers’ health (Mititelu et al. 2022). Lead concentrations in the A1 area proved to be above acceptable levels, with the highest concentrations being detected in propolis and multifloral honey samples. The same case was noticed regarding the concentrations of zinc, detected in high amounts in polyfloral honey and propolis samples. Comparatively, in several situations, high concentrations of lead modified the feeding behavior of bees and altered their sensitivity to sucrose (Burden et al. 2019). Despite the fact that zinc is an essential mineral, high bioaccumulation levels can result in a number of unpleasant symptoms, including nausea, vomiting, diarrhea, fever, and tiredness. Conversely, significantly lower levels of heavy metals were detected in bee products collected from Area 2 (Mititelu et al. 2022). Therefore, beekeepers should pay attention to the locations of their apiaries, whereas consumers should check the sources of acquired bee products.

## Toxic compounds in bee products

Among the potential contaminants found in bee products, there are pesticides, metals, pyrrolizidine alkaloids, and mycotoxins. Since scientific data on the mycotoxin content in bee products in particular is lacking, further studies are reportedly needed to establish food safety hazards (Végh et al. 2021).

Pyrrolizidine alkaloids (PA) are secondary metabolites of plants, mostly found in the genera *Crotalaria*, *Echium*, *Eupatorium*, and *Senecio*. The presence of 1,2-unsaturated PA in foods is a major risk listed by food regulators around the world. This may be due to the fact that these compounds have been associated with acute and chronic toxicity, especially in relation to liver health. The intake of these nutrients usually occurs through the accidental ingestion of plant material and derivatives, as well as products of plant or animal origin, such as honey. PA or PA *N*-oxide in nectar, honeydew, and pollen collected from the flora by honey bees are transferred to honey (Brugnerotto et al. 2021).

A recent study determined the presence of low levels of PAs in Italian honey samples; specifically, 35 PAs were detected, ranging from 0.9 μg/kg to 33.1 μg/kg, with echimidine being the most prevalent in polyfloral honey, followed by *Castanea*. A rapid human exposure assessment of PAs in honey and a risk characterization using the EFSA RACE tool were reported. The assessment indicated that there may be a potential health concern only for children who frequently consume high quantities of honey (Roncada et al. 2023). Furthermore, echimidine has been associated with immune-related inflammation and intestinal toxicity, that may lead to gastrointestinal discomfort, including diarrhea and stomachache (Ru et al. 2019).

One of the food safety concerns related to honey is the fact that honey bees collect nectar from poisonous plants such as *Rhododendron* sp., *Coriaria arborea*, and *Tripterygium wilfordii*. These types of honey contain natural plant toxins, such as grayanotoxins, triptolides, tutin, and pyrrolizidine alkaloids (Fig. 2). Triptolids are known to cause poisoning symptoms and, in several cases, even death after ingestion. Triptolide toxicity is dosage- and time-dependent, mostly affecting the heart, kidney, liver, spleen, circulatory and gastrointestinal systems, and reproductive organs (Li et al. 2014b). However, in rare cases, this compound is used as an immunosuppressive and anti-cancer treatment (Song et al. 2023). Although different toxic honeys produce similar symptoms, such as vomiting, nausea, and dizziness, the mechanism of toxicity has been reported to be different (DiSalvo et al. 2022; Islamoglu et al. 2021; Lucatello et al. 2022; Yan et al. 2022).

Alkaloids found in honey produced from *Rhododendron ponticum* nectar can be poisonous to humans, whereas grayanotoxins found in honey blossoms from *Andromeda* species can paralyze limbs, cause hypotension, bradycardia, and eventually lead to death (Aygun et al. 2018). In addition, New Zealand produces poisonous honey from *Melicope ternata* and *Coriaria arborea*, which can be lethal. There is evidence that honey is not safe for consumption when collected from *Datura* plants (Mexico and Hungary), *Atropa belladonna* flowers and *Hyoscamus niger* plants (Hungary), *Serjania lethalis* (Brazil), and *Gelsemium sempervirens* (USA). Although the signs of honey poisoning vary according to the origin of the toxins, the most typical signs are often lightheadedness, nausea, vomiting, convulsions, headaches, and palpitations, which lead to a higher chance of mortality (Islam et al. 2014; Yan et al. 2022).

## Improper storage of bee products

The development of certain microorganisms requires a suitable substrate, such as BCP, due to its high moisture content and elevated water activity, which promotes the quick growth of fungi and induces the production of mycotoxins (Kostić et al. 2019). Toxigenic fungi are particularly risky among these microorganisms due to their ability to produce mycotoxins as part of their metabolic processes. In addition, mycotoxins are frequently discovered during pollen formation and/or collecting process (e.g., even at ideal temperature, relative humidity, pH, and water activity levels). Isolated mycotoxins from BCP samples include aflatoxins, deoxynivalenol, fumonisins, ochratoxins, zearalenone, and T-2 toxin, with a notable prevalence (Kostić et al. 2019). BCP stored for longer periods led to the formation of toxins such as zearalenone (65–280 μg/kg) and deoxynivalenol (47–120 μg/kg), which are produced by various *Fusarium* species. A positive correlation was found between the moisture content of BCP and zearalenone formation. Conversely, no correlation was found between storage duration or the total amount of fungi and the formation of zearalenone and deoxynivalenol (Sinkevičienė et al. 2023). It is crucial to investigate different mycotoxins in BCP samples presented to the consumer, to determine possible aflatoxin transmission routes, and to make regular mycotoxicological analyses of the pollen. More details related to pathogens in bee products can be found in Table 3.

**Table 3:** Examples of detected pathogen risks in bee products, such as honey and pollen

Country	Detected pathogen	Bee product	References
Slovakia	*Aspergillus flavus*, *A. parasiticus*, *Fusarium graminearum*	BCP	Kačániová et al. 2011
Spain & Argentina	*A. flavus*, *A. parasiticus*	BCP	González et al. 2005
Bulgarian	*Aspergillus* spp., *Fusarium* spp., *Penicillium* spp., *Alternaria* spp. *Cladosporium* spp., other species;	fresh and dry BCP	Beev et al. 2018
Italy	*Cladosporium* spp., *Alternaria* spp, *Humicola* spp., Mucoraceae, *Acremonium* spp., *Penicillium* spp., *Aspergillus* spp.	BCP	Nardoni et al. 2016
Japan	*Clostridium botulinum*	Honey	Nakano and Sakagucki 1991
Finland	*Clostridium botulinum*	Honey	Nevas et al. 2002

Note: *BCP* bee-collected pollen

Deoxynivalenol (DON), HT-2 toxin (HT2), T-2 toxin (T2), and ochratoxin A (OTA) concentrations in bee products (BCP, propolis, honey, and royal jelly) were measured, along with evaluations of exposure and risk, to determine the possible negative impact on health. DON and T-2 toxin, with average values of 1.6 and 0.7 μg/kg dry samples, were the most prevalent mycotoxins in all bee products. According to reports, mycotoxins ingested as a result of consuming bee products in certain proportions do not cause a health concern (Keskin and Eyupoglu 2023).

Food packaging often uses a variety of plastics. Food samples, particularly honey products, usually include evidence of plastic migration. The numerous varieties of honey are categorized according to viscosity, pH, and moisture content. Plastic containers deteriorate at a different rate depending on the temperature, humidity, and light of the storage environment. Given that it may be preserved for an extended period of time (Ünal et al. 2010), honey in particular has the potential to degrade its plastic packaging more quickly. In order to completely minimize the risk of MP/NP migration, plastic packaging usage should be reduced. Moreover, new packaging materials that are completely resistant to the aforementioned stress conditions should be developed. Thus, consumers are advised to pay particular attention to how products are packed and how they are stored (Katsara et al. 2022).

## Future perspectives

When talking about the detrimental effects of environmental pollution factors on bee populations and bee products, it is also important to discuss promising future perspectives using a multifaceted approach. On one hand, organic and sustainable farming methods will need to be included in agricultural practices, reducing the reliance on the abovementioned synthetic pesticides and fertilizers (Pocol et al. 2021; Wintermantel et al. 2019). On the other hand, awareness regarding this topic might play a crucial role in gathering public engagement and encouraging individuals to adopt bee-friendly practices, worth mentioning being the cultivation of pollinator-friendly green spaces (Durant and Ponisio 2021; Hipólito et al. 2016). Furthermore, the potential of bees and their products to serve as valuable bioindicators of environmental pollution should be considered (Bargańska et al. 2016; Costa et al. 2019; Traynor et al. 2021).

The use of ecofriendly insecticides (i.e., essential oils) to counteract the negative effects of organosynthetic insecticides on honey bees’ wellbeing and to control pest infestations proves to be an effective and innovative treatment. Application of Negramin (*Siparuna guianensis* Aubl) EO exhibited repellent activity against both *Achroia grisella* F. and *Galleria mellonella* L. wax moths, without compromising the foraging activities of honey bees (Ferreira et al. 2017). Compared to commercial insecticides (i.e., imidacloprid), major compounds obtained from *Lippia sidoides* (rosemary pepper) EO exhibit low lethal toxicity (LD_50_ = 306 μg/bee) compared to organosynthetics (LD_50_ = 0.001−0.009 μg/bee) (Matos et al. 2021). Furthermore, a mixture of *Thymus satureioides* and *Origanum elongatum* significantly reduces the infestation rate (94%) with *V. destructor*, with no reported negative effects on honey bees (Ramzi et al. 2017). Future studies may focus on the development of nanoemulsions or encapsulations to increase EO bioavailability and enhance their effectiveness.

## Conclusions

The production of reliable bee products for a healthy society is the primary duty of all beekeepers. In order not to cause residue, pesticides should be used as little as possible during the flowering period and remain completely unused, especially during the feeding flights of the honey bees. Beekeepers should not forget that they can reduce the risk of residues by moving their hives at least 3 km away from pesticide-treated agricultural areas. As a result of thorough inspections of agricultural organizations, excessive and improper use of pesticides should be controlled by professionals. The use of biological pest control methods by farmers is mandatory, including the use of natural predators against pests and parasites. If chemical spraying is mandatory, it is beneficial to choose between biological and botanical derivatives and alternatives to new pesticides that are believed to reduce honey bee and human health and environmental risks. Honey monitoring programs should be established and maintained to ensure the consumer’s risk-free use of honey. Furthermore, there is a critical need for more studies that can accurately analyze the hidden aspects of this issue and discover appropriate remedial measures. Finally, it is important to regulate bee pollen as a dietary supplement and to set recommended tolerable limits for certain mycotoxins, due to the fact that the microbiological quality of the bee pollen is as important as its nutritional properties.

## Data Availability

Data sharing is not applicable.
